# A numerical study of the European option by the MLPG method with moving kriging interpolation

**DOI:** 10.1186/s40064-016-1947-5

**Published:** 2016-03-09

**Authors:** P. Phaochoo, A. Luadsong, N. Aschariyaphotha

**Affiliations:** Department of Mathematics, Faculty of Science, King Mongkut’s University of Technology Thonburi (KMUTT), 126 Pracha-utid Road, Bangmod, Toongkru, Bangkok, 10140 Thailand; Ratchaburi Learning Park, King Mongkut’s University of Technology Thonburi (KMUTT), Rang Bua, Chom Bueng, Ratchaburi 70150 Thailand

**Keywords:** Black–Scholes equation, European option, MLPG, Moving kriging interpolation, Stability

## Abstract

In this paper, the meshless local Petrov–Galerkin (MLPG) method is applied for solving a generalized Black–Scholes equation in financial problems. This equation is a PDE governing the price evolution of a European call or a European put under the Black–Scholes model. The *θ*-weighted method and MLPG are used for discretizing the governing equation in time variable and option pricing, respectively. We show that the spectral radius of amplification matrix with the discrete operator is less than 1. This ensures that this numerical scheme is stable. Numerical experiments are performed with time varying volatility and the results are compared with the analytical and the numerical results of other methods.

## Background

The Black–Scholes equation, which is proposed by Black and Scholes (1973), is a financial model that is concerned with options. An option is a contract between the seller and the buyer. It consists of a call option and a put option. Option valuation depends on the underlying asset price and time. European options can only be exercised at the expiration date, but American options can be exercised at any time before the expiration date. The Black–Scholes equation provides an option pricing formula for European option. The analytic solution is used in general cases with basic assumptions but it is not satisfied in some conditions.

The numerical methods for solving the Black–Scholes equation have been presented in many scholarly studies. The binomial process and probability for formula valuation of option pricing are proposed by Cox et al. The rate of return on the stock over each period can have two possible values and the end stock price of maturity will be either *us* or *ds* (Cox et al. 1979). Moon and Kim use an adaptive averaging binomial method for option valuation (Moon and Kim 2013). The symmetrical lattice for real options valuation are presented by Bastian-Pinto (2015). Glazyrina and Melnikov presented an alternative derivation of the Black–Scholes formula from a binomial option pricing model (Glazyrina and Melnikov 2016). The finite difference method by Schwartz developed the numerical solution for valuing options on dividend-paying stocks. The boundary condition is enforced to take into account the fact that the stock price will drop by the amount of the dividend (Schwartz 1977). A numerical upwind scheme for solving the backward time parabolic partial differential equation is proffered by Vazquez (1998). A robust finite difference method for pricing European and American options is presented by Cen and Le (2010, 2011). Cen et al. introduced a central difference scheme with moving mesh in the spatial discretization for pricing Asian options (Cen et al. 2013). Lesmana and Wang purposed an upwind finite difference method for a nonlinear Black–Scholes equation (Lesmana and Wang 2013). The uniform cubic B-spline collocation method is implemented to find the numerical solution by a horizontal method of lines, to discretize the temporal variable and the spatial variable (Kadalbajoo et al. 2012).

Kumar et al. suggested that the governing equation is discretized by the *θ*-weighted method and the option price is approximated by the radial basis functions based on the finite difference method (Kumar et al. 2015). Mohammadi presented Quintic B-spline collocation approach for solving generalized Black–Scholes equation governing option pricing, the horizontal method of lines for time integration and *θ*-method are used for temporal discretization (Mohammadi 2015). The fractional Black–Scholes model are presented by Bjork and Hult (2005), Song and Wang (2013). Kumar et al. presented a numerical computation of fractional Black–Scholes equation arising in financial market (Kumar et al. 2014). The meshless local Petrov–Galerkin (MLPG) method based on moving kriging interpolation for solving fractional Black–Scholes model are purposed by Phaochoo et al. (2016). Kleinert and Korbel presented double-fractional differential equation for the prices of options (Kleinert and Korbel 2016). Zhang et al. introduced the numerical simulation of the tempered fractional Black–Scholes equation for European double barrier option (Zhang et al. 2016).

A truly meshless method for solving boundary value problems based on the local symmetric weak form and the moving least squares (MLS) approximation, called the MLPG method, has been proposed and successfully applied to solve many problems (Atluri and Shen 2002). The MLPG method is one of the most viable methods in which the moving least square (MLS) approach is used to construct the shape functions. Although the MLPG method has been applied to many problems, there exists an inconvenience or disadvantage when using the MLPG because of the difficulty in implementing essential boundary conditions. This is because the MLS shape functions lack the Kronecker delta property. Therefore the moving kriging interpolation (MKI) method (Yimnak and Luadsong 2014) has been proposed to overcome this problem. It uses nodal values in the local support domain to construct shape functions with the Kronecker delta property. The MKI method works well for practical problems.

In this paper, we propose a numerical method based on the MLPG method to solve a generalized Black–Scholes equation. The MLPG is a truly meshless method, which involves not only a meshless interpolation for the trial functions, but also a meshless integration of the weak-form. MLPG type 2 (MLPG2) is chosen for this research so the Kronecker delta is the test function. This method will avoid the domain integral in the weak-form. In addition, we compared numerical solutions among the finite difference method, the cubic spline method and the MLPG method in the numerical experiment.

## Problem formulation

The Black–Scholes equation is the outstanding financial equation that solves European option pricing without a transaction cost. Moreover, underlying asset prices distributed on the log indicate normal random walks, risk-free interest rates, no dividends and no arbitrate opportunities as fundamental assumptions. The Black–Scholes equation follows:

1 $$\frac{\partial u}{\partial \tau } + r(\tau )s\frac{\partial u}{\partial s} + \frac{1}{2}\sigma^{2} (s,\tau )s^{2} \frac{{\partial^{2} u}}{{\partial s^{2} }} - r(\tau )u = 0, \quad (s,\tau ) \in {\mathbb{R}}^{ + } \times [0,T]$$

with terminal and boundary conditions

$$u(s,T) = \hbox{max} (s - E,0), s \in {\mathbb{R}}^{ + } ,\;\;u(0,\tau ) = 0,\tau \in [0,T],$$

where *u*(*s*, *τ*), *T*, *r* and σ are the value of European call options at underlying asset price *s* at time *τ,* the expiration date, risk-free interest rate and volatility of underlying asset prices, respectively. From Eq. (1), when *s* goes to zero then degenerating will occur in approximation. We transform the Black–Scholes equation into a non-degenerate partial differential equation by using a logarithmic transformation, *x* = ln *s*, *t* = *T* − *τ*, and define the computational domain for convenience in numerical experiments by Ω = [*x*_min_, *x*_max_] × [0, *T*], where *x*_min_ = −ln(4*E*), *x*_max_ = ln(4*E*) (Huang and Cen 2014).

2 $$\frac{\partial u}{\partial t} = \frac{1}{2}\sigma^{2} (x,t)\frac{{\partial^{2} u}}{{\partial x^{2} }} + \left( {r(t) - \frac{1}{2}\sigma^{2} (x,t)} \right)\frac{\partial u}{\partial x} - r(t)u,\quad (x,t) \in \Omega$$

$$\begin{aligned} u(x,0) & = \hbox{max} \left( {e^{x} - E,0} \right),x \in \left( {x_{\hbox{min} } ,x_{\hbox{max} } } \right), \\ u\left( {x_{\hbox{min} } ,t} \right) & = 0,\;\;u\left( {x_{\hbox{max} } ,t} \right) = e^{{x_{\hbox{max} } }} - Ee^{{ - \int_{0}^{t} {r(s)ds} }} ,\quad t\in \left[ {0,T} \right]. \\ \end{aligned}$$

## Moving kriging interpolation

The moving kriging interpolation (MKI) is used to construct the shape function. The function *u*(*x*) is defined in the domain Ω and the approximate function is *u*^*n*^(*x*). The subdomain Ω_x_ that encompasses these surrounding nodes is called the interpolation domain of point *x*. The formulation of the meshless shape function using MKI is given by

3 $$u^{h} (x) = \mathop \sum \limits_{j = 1}^{N} \phi_{j} \hat{u}_{j} (t) = \varPhi (x)U(t),\quad x \in \Omega ,$$

where $U(t) = \left[ {\hat{u}_{1} (t)\hat{u}_{2} (t)\hat{u}_{3} (t) \ldots \hat{u}_{N} (t)} \right]^{T}$ is a vector value of the function in the domain Ω. Φ(*x*) is a 1 × *N* vector of shape functions, expressed as

4 $$\Phi (x) = P^{T} (x)A + r^{T} (x)B,$$

where matrix *A* and *B* are defined as

5 $$A = \left( {P^{T} R^{ - 1} P} \right)^{ - 1} P^{T} R^{ - 1} ,$$

6 $$B = R^{ - 1} \left( {I - PA} \right),$$

and

7 $$p^{T} (x) = \left[ {p_{1} (x_{1} )p_{2} (x_{2} )p_{3} (x_{3} ) \ldots p_{m} (x_{N} )} \right].$$

For matrix *P* with size *N* × *m*, values of the polynomial basis function Eq. (7) at the given set of nodes are collected as follows

8 $$P = \left[ {\begin{array}{*{20}c} {p_{1} (x_{1} )} & \cdots & {p_{m} (x_{1} )} \\ \vdots & \ddots & \vdots \\ {p_{1} (x_{N} )} & \cdots & {p_{m} (x_{N} )} \\ \end{array} } \right].$$

Matrix *R* and vector *r*(*x*) are defined by the following

9 $$P = \left[ {\begin{array}{*{20}c} {\gamma (x_{1} ,x_{1} )} & \cdots & {\gamma (x_{1} ,x_{1} )} \\ \vdots & \ddots & \vdots \\ {\gamma (x_{N} ,x_{1} )} & \cdots & {\gamma (x_{N} ,x_{N} )} \\ \end{array} } \right],$$

10 $$r^{T} (x) = \left[ {\gamma \left( {x,x_{1} } \right)\;\gamma \left( {x,x_{2} } \right)\; \ldots \;\gamma \left( {x,x_{N} } \right)} \right],$$

where *γ*(*x*_*i*_, *x*_*j*_) is the correlation between any pair of nodes located at *x*_*i*_ and *x*_*j*_, representing the covariance of the field value *u*(*x*). A simple and frequently used correlation function is a Gaussian function as

11 $$\gamma \left( {x_{i} ,x_{j} } \right) = e^{{ - \epsilon r_{ij}^{2} }} ,$$

where $r_{ij} = \left\| {x_{i} - x_{j} } \right\|$ and $\epsilon > 0$ are the correlation and shape parameters, respectively used to fit the model.

## Spatial discretization

The MLPG method constructs the local weak form over the local subdomain, which is a small region taken for each node in a global domain. Multiplying test function *v*_*i*_(*x*) into Eq. (2) and then integrating it over subdomain $\Omega_{s}^{i}$ yields the following expression:

12 $$\int_{{\Omega_{s}^{i} }} {\frac{\partial u}{\partial t}v_{i} \left( x \right)d\Omega } = \int_{{\Omega_{s}^{i} }} {\left( {\frac{1}{2}\sigma^{2} (x,t)\frac{{\partial^{2} u}}{{\partial x^{2} }} + \left( {r(t) - \frac{1}{2}\sigma^{2} (x,t)} \right)\frac{\partial u}{\partial x} - r(t)u} \right)} v_{i} (x)d\Omega ,$$

where *v*_*i*_ is a test function that is significant for each node. Rearranging Eq. (12), we have

13 $$\begin{aligned} \int_{{\Omega_{s}^{i} }} {\frac{\partial u}{\partial t}v_{i} (x)d\Omega }& = \frac{1}{2}\int_{{\Omega_{s}^{i} }} {\sigma^{2} (x,t)u,_{xx} v_{i} (x)d\Omega }\\&\quad + \int_{{\Omega_{s}^{i} }} {\left( {r(t) - \frac{1}{2}\sigma^{2} (x,t)} \right)u,_{x} v_{i} (x)d\Omega } \\&\quad- \int_{{\Omega_{s}^{i} }} {r(t)uv_{i} (x)d\Omega } , \end{aligned}$$

where $u,_{xx} = \frac{{\partial^{2} u}}{{\partial x^{2} }},u,_{x} = \frac{\partial u}{\partial x}$. Substituting trial function $u^{h} (x,t) = \sum\nolimits_{j = 1}^{N} {\phi_{j} (x)\hat{u}_{j} (t)}$ into *u* and its derivative in Eq. (13)

14 $$\begin{aligned} \int_{{\Omega_{s}^{i} }} {\mathop \sum \limits_{j = 1}^{N} \phi_{j} (x)v_{i} (x)\frac{{d\hat{u}_{j} }}{dt}d\Omega } & = \frac{1}{2}\int_{{\Omega_{s}^{i} }} {\mathop \sum \limits_{j = 1}^{N} \phi_{j,xx} (x)\sigma^{2} (x,t)v_{i} (x)\hat{u}_{j} d\Omega } \\ & \quad + \int_{{\Omega_{s}^{i} }} {\mathop \sum \limits_{j = 1}^{N} \phi_{j,x} (x)\left( {r(t) - \frac{1}{2}\sigma^{2} (x,t)} \right)v_{i} (x)\hat{u}_{j} d\Omega } \\ & \quad - \int_{{\Omega_{s}^{i} }} {\mathop \sum \limits_{j = 1}^{N} \phi_{j} (x)r(t)v_{i} (x)\hat{u}_{j} d\Omega } , \\ \end{aligned}$$

where *N* is the number of nodes surrounding point *x* which has an effect on *u*(*x*) and $\hat{u}_{j} (t)$ is value of the option at time *t*. The shape function, *ϕ*_*j*_(*x*), is constructed by the moving kriging interpolation which has the Kronecker delta property, thereby enhancing arrangement of the nodal shape construction accuracy. Rearranging Eq. (14) yields the following results:

15 $$\begin{aligned} \sum\limits_{j = 1}^{N} {\int_{{\Omega_{s}^{i} }} {\phi_{j} (x)v_{i} (x)d\Omega \frac{{d\hat{u}_{j} }}{dt}} } & = \frac{1}{2}\sum\limits_{j = 1}^{N} {\int_{{\Omega_{s}^{i} }} {\phi_{j,xx} (x)\sigma^{2} (x,t)v_{i} (x)d\Omega \hat{u}_{j} } } \\ & \quad + \mathop \sum \limits_{j = 1}^{N} \int_{{\Omega_{s}^{i} }} {\phi_{j,x} \left( x \right)\left( {r\left( t \right) - \frac{1}{2}\sigma^{2} \left( {x,t} \right)} \right)v_{i} \left( x \right)d\Omega \hat{u}_{j} } \\ & \quad - \mathop \sum \limits_{j = 1}^{N} \int_{{\Omega_{s}^{i} }} {\phi_{j} \left( x \right)r\left( t \right)v_{i} \left( x \right)d\Omega \hat{u}_{j} } , \\ \end{aligned}$$

This research uses MLPG type 2 (MLPG2) (Cen and Le 2011), then the test function *v*_*i*_ is chosen by the Kronecker delta function,

$$v_{i} (x) = \left\{ {\begin{array}{*{20}l} {0}, &\quad {x \ne x_{i} } \hfill \\ {1}, &\quad {x = x_{i} } \hfill \\ \end{array} ,\quad i = 1,2, \ldots ,N.} \right.$$

The test function will define significance for each node in the subdomain. In this case, substituting test function *v*_*i*_(*x*) to Eq. (15) and then integrating it over subdomain $\Omega_{s}^{i}$ yields the following results:

16 $$\begin{aligned} \sum\limits_{{j = 1}}^{N} {\phi _{j} } \left( {x_{i} } \right)\frac{{d\hat{u}_{j} }}{{dt}} & = \sum\limits_{{j = 1}}^{N} {\left[ {\frac{1}{2}\sigma ^{2} \left( {x_{i} ,t} \right)\phi _{{j,xx}} \left( {x_{i} } \right)} \right.} \\ & \quad \left. { + \left( {r(t) - \frac{1}{2}\sigma ^{2} \left( {x_{i} ,t} \right)} \right)\phi _{{j,x}} \left( {x_{i} } \right) - r(t)\phi _{j} \left( {x_{i} } \right)} \right]\hat{u}_{j} . \\ \end{aligned}$$

Equation (16) can be written in matrix form as follows:

17 $$A\frac{dU}{dt} = BU,$$

where

$$\begin{aligned} A & = \left[ {A_{ij} } \right]_{N \times N,} \quad A_{ij} = \phi_{j} \left( {x_{i} } \right), \\ B & = \left[ {B_{ij} } \right]_{N \times N} ,\quad B_{ij} = \frac{1}{2}\sigma^{2} \left( {x_{i} ,t} \right)\phi_{j,xx} \left( {x_{i} } \right) + \left( {r(t) - \frac{1}{2}\sigma^{2} \left( {x_{i} ,t} \right)} \right)\phi_{j,x} \left( {x_{i} } \right) - r(t)\phi_{j} \left( {x_{i} } \right)], \\ U & = \left[ {\hat{u}_{1} \hat{u}_{2} \hat{u}_{3} \ldots \hat{u}_{N} } \right]^{T} . \\ \end{aligned}$$

Since the shape function that is constructed by the moving kriging interpolation satisfies the Kronecker delta property, *A* is the identity matrix. Therefore, Eq. (17) can be written as

18 $$\frac{dU}{dt} = BU.$$

## Temporal discretization

The numerical solution of a European option, using the implicit method, requires the generation of a modified PDE operator through a finite difference approximation of time derivative. We will do this using the *θ*-weighted method.

Consider the following initial-boundary value problem:

$$\frac{{\partial u\left( {x,t} \right)}}{\partial t} = {\mathcal{L}}u(x,t),\quad x \in \Omega ,0 \le t \le T,$$

$$u(x,0) = u_{0} ,$$

19 $$u(x,t) = g(x,t),\quad x \in \partial \Omega_{x}$$

where $${\mathcal{L}}u = \frac{1}{2}\sigma^{2} \left( {x,t} \right)\frac{{\partial^{2} u}}{{\partial x^{2} }} + \left( {r(t) - \frac{1}{2}\sigma^{2} \left( {x,t} \right)} \right)\frac{\partial u}{\partial x} - r(t)u.$$

By a finite difference approximation made for the time derivative with notation *u*^*n*^(*x*) that approximates the exact solution *u*(*x*, *t*) at *t*^*n*^ and *t*^*n*^ = *t*^*n*−1^ + Δ*t*, we obtain

20 $$\frac{{u^{n + 1} - u^{n} }}{\Delta t} = \theta {\mathcal{L}}u^{n + 1} + \left( {1 - \theta } \right){\mathcal{L}}u^{n} .$$

For each fixed time level *t*_*n*_, the above equation, Eq. (20), is the system of linear ODEs. Now, using the MLPG2 method with moving kriging interpolation for constructing shape functions is discussed through Eqs. (12)–(18) for spatial discretization of operator ${\mathcal{L}}u$ leads to

21 $$\left( {I - \theta \Delta tB} \right)U^{n + 1} = \left[ {I + \left( {1 - \theta } \right)\Delta tB} \right]U^{n} ,$$

where $U^{n} = \left[ {\hat{u}_{1}^{n} \hat{u}_{2}^{n} \hat{u}_{3}^{n} \ldots \hat{u}_{N}^{n} } \right]^{T} ,$*B* is the discretization matrix for the space discretization of linear differential operator ${\mathcal{L}}u$, and *I* is the identity matrix.

## Stability analysis

In this section, we present an analysis of the stability of the MLPG2 method with moving kriging interpolation using the matrix method. A small fluctuation at the *n*th time level $e^{n} = U^{n} - \tilde{U}^{n}$ is introduced in Eq. (21), where *U*^*n*^ is exact and $\tilde{U}^{n}$ is the numerical solution. The equation of the error *e*^*n*+1^ can be written as *e*^*n*+1^ = *Ge*^*n*^, where the amplification matrix *G* = [*I* − *θ*∆*tB*]^−1^ [*I* + (1 − *θ*)∆*tB*]. The numerical scheme will be stable if as $n \to \infty ,$ the error $e^{n} \to 0$. This can ensure that *ρ*(*G*) < 1 provided, where *ρ*(*G*) denotes the spectral radius of *G*.

**Fig. 1:**
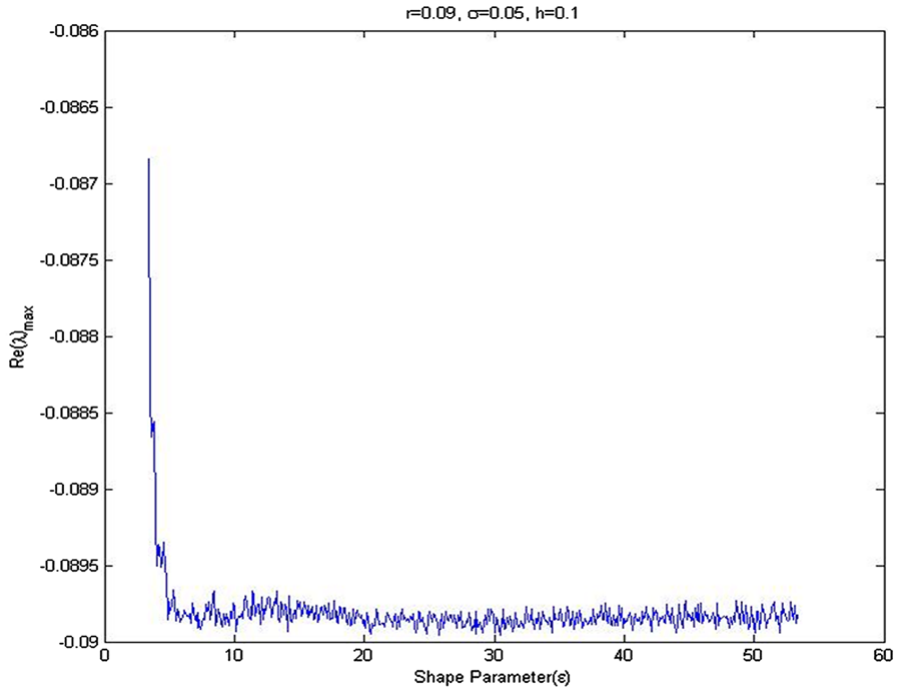
The relation between *Re*(*λ*)_max_ and shape parameter ($\epsilon$)

It can be seen that stability is assured if all eigenvalues of the matrix [*I* − *θ*∆*tB*]^−1^ [*I* + (1 − *θ*)∆*tB*] satisfy the following conditions:

22 $$\left| {\frac{{1 + \left( {1 - \theta } \right)\Delta t\lambda }}{1 - \theta \Delta t\lambda }} \right| \le 1$$

where *λ* is the eigen value of matrix *B*. In the case of the Crank–Nicolson scheme ($\theta = \frac{1}{2}$) the inequality in Eq. (22) is satisfied when *Re*(*λ*)_*max*_ ≤ 0. This shows that the scheme is stable if *Re*(*λ*) ≤ 0. The eigenvalues of matrix *B* highly depends on the mesh spacing parameter *h* and the shape parameter $\epsilon$, where *h* is defined to be the minimal distance between any two points in the domain. Since it is not possible to find an explicit relationship among the eigenvalue of matrix *B*, the number of nodes and the shape parameter $\epsilon$ we investigated this dependent numerically, as is given in Fig. 1.

**Fig. 2:**
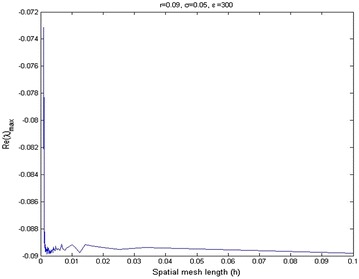
Relation between *Re*(*λ*)_max_ and spatial mesh length (*h*)

**Fig. 3:**
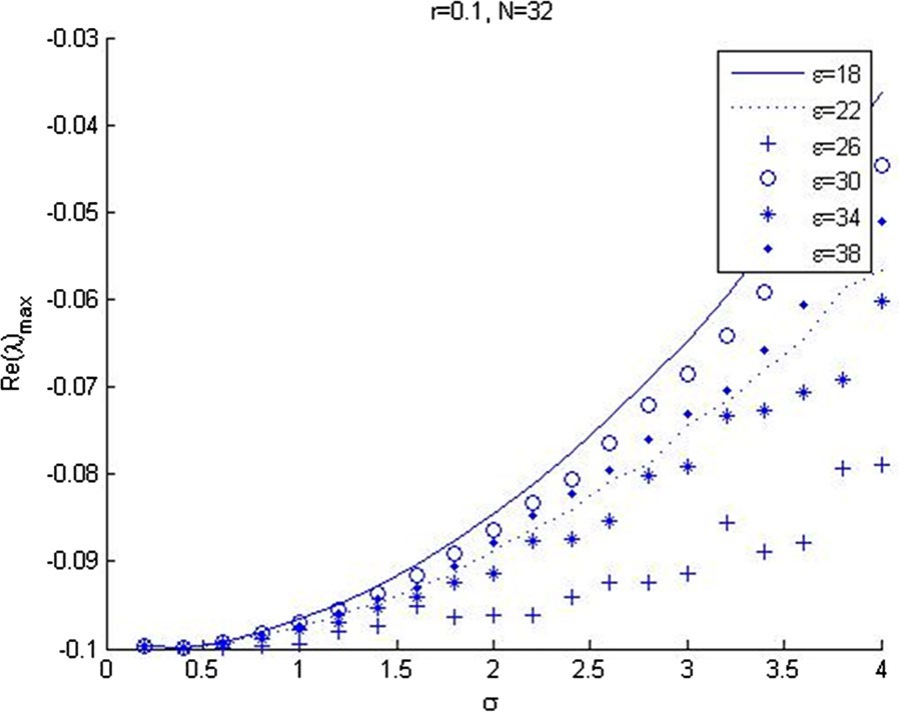
The relation between *Re*(*λ*)_max_ and the volatility (*σ*)

**Fig. 4:**
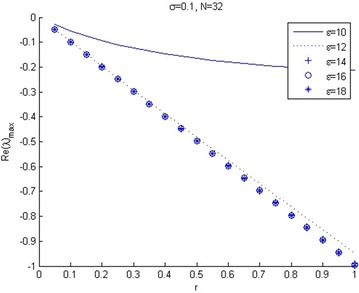
The relation between *Re*(*λ*)_max_ and risk free interest rate (*r*)

**Fig. 5:**
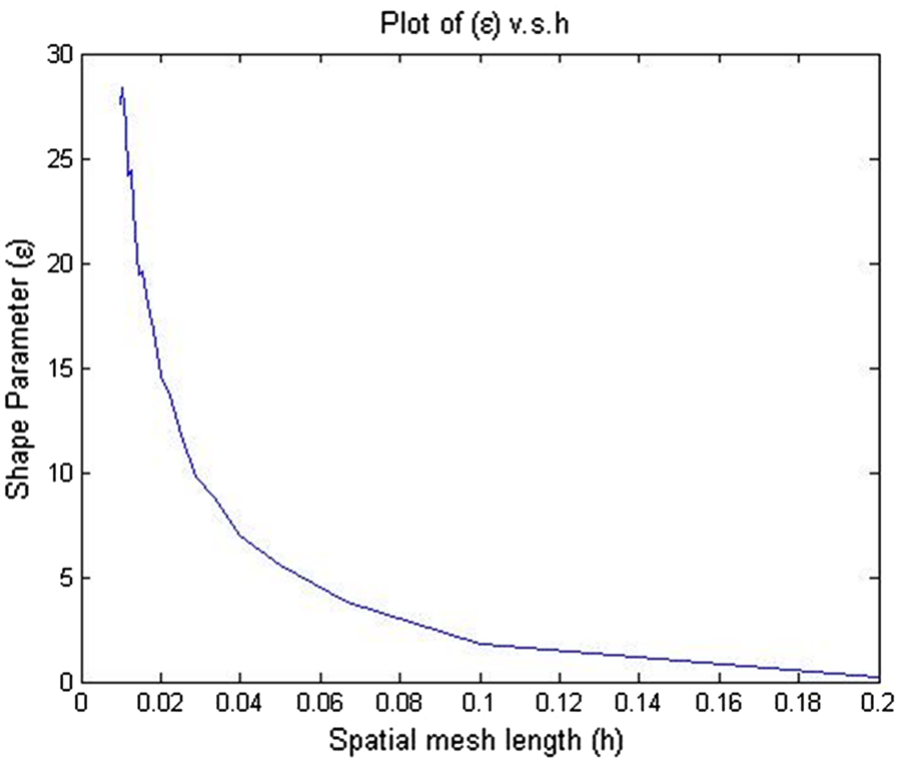
The relation between the smallest of shape parameter *ϵ* and the mesh length ‘*h*’

Figure 1 shows that the maximum eigenvalue *Re*(*λ*) of matrix *B* varies as a function of shape parameter $\epsilon$, when mesh spacing parameter *h* is constant. Figure 2 shows the effect of mesh length, *h*, for eigenvalue of matrix *B*, when the shape parameter $\epsilon$ is constant. It is found that the condition number of the collocation matrix becomes very large and the system leads to ill-conditioning, when $\epsilon$ and *h* become very small. Figure 3 shows that increasing volatility trends to ill-conditioning. In this case, if the shape parameter increases then eigenvalue *Re*(*λ*) will decrease. Figure 4 presents that a risk-free interest rate which decreases leads to ill-conditioning and if the shape parameter increases, then eigenvalue *Re*(*λ*) will decrease. Figure 5 shows the relation between mesh length *h* and the smallest of shape parameter $\epsilon$ is *Re*(*λ*)_*max*_ < 0. It shows that $\epsilon$ could be large when *h* becomes smaller in value. It can be seen that we can control the stability of this numerical scheme by choosing the appropriate shape parameter.

## Numerical experiments

In this section, we are going to present various numerical results to evaluate proposed meshless approaches. Although the schemes work for all correlation functions, we will use the Gaussian function on different experimental setups. Using the MLPG2 method, the resulting problems for European call options are solved via Crank–Nicolson’s method. The computational domain is partitioned with *N* being equi-spaced spatial nodes with mesh lengths. The temporal domain is divided into *K* equi-spaced points.

### The European call option can be modeled by the Black–Scholes PDE

23 $$\frac{\partial u}{\partial \tau } + r\left( \tau \right)s\frac{\partial u}{\partial s} + \frac{1}{2}\sigma^{2} \left( {s,\tau } \right)s^{2} \frac{{\partial^{2} u}}{{\partial s^{2} }} - r\left( \tau \right)u = 0$$

with the payoff function given by

24 $$u\left( {s,T} \right) = \hbox{max} \left( {s - E,0} \right),\quad s \in {\mathbb{R}}^{ + } .$$

The boundary conditions are given as

25 $$u\left( {0,\tau } \right) = \left\{ {\begin{array}{*{20}l} {0}, &\quad {s = 0} \\ {s - Ee^{{ - r\left( {T - \tau } \right) }} }, &\quad {s \to \infty } \hfill \\ \end{array} } \right..$$

The analytical solution for the European call option is

26 $$u\left( {s,\tau } \right) = sN\left( {d_{1} \left( {s,\tau } \right)} \right) - Ee^{{ - r\left( {T - \tau } \right)}} N\left( {d_{2} \left( {s,\tau } \right)} \right)$$

where *N*(·) is the cumulative distribution function of the standard normal distribution with

$$\begin{aligned} d_{1} \left( {s,\tau } \right) & = \frac{{\ln \left( {\frac{s}{E}} \right) + \left( {r + \left( {\frac{1}{2}} \right)\sigma^{2} } \right)\left( {T - \tau } \right)}}{{\sigma \sqrt {T - \tau } }}, \\ d_{2} \left( {s,\tau } \right) & = \frac{{\ln \left( {\frac{s}{E}} \right) + \left( {r - \left( {\frac{1}{2}} \right)\sigma^{2} } \right)\left( {T - \tau } \right)}}{{\sigma \sqrt {T - \tau } }}. \\ \end{aligned}$$

A simple transformation *x* = ln *s* and *t* = *T* − *τ* transforms Eq. (23) and conditions (24)–(25) to

27 $$\frac{\partial u}{\partial t} = \frac{1}{2}\sigma^{2} (x,t)\frac{{\partial^{2} u}}{{\partial x^{2} }} + \left( {r(t) - \frac{1}{2}\sigma^{2} (x,t)} \right)\frac{\partial u}{\partial x} - r(t)u, \quad (x,t) \in \Omega$$

with initial and boundary conditions

28 $$u(x,0) = \hbox{max} \left( {Ee^{x} - E,0} \right)$$

29 $$u(x,t) = \left\{ {\begin{array}{*{20}l} {0}, &\quad {x \to - \infty } \hfill \\ {Ee^{x} - Ee^{ - rt }}, &\quad {x \to \infty } \hfill \\ \end{array} .} \right.$$

To illustrate accuracy of the proposed method, numerical simulation was done for the European call option with parameters σ = 0.4, *r* = 0.8, *T* = 1, *E* = 1, *x*_min_ = −ln(4*E*), *x*_max_ = ln(4*E*). Accuracy is measured in the discrete maximum norm and root mean square error. The discrete maximum norm and maximum of root mean square error are given in Table 1 that is estimated for differences *N* and *K* in the MLPG2 methods.

$$E_{\infty }^{N} = \mathop {\hbox{max} }\limits_{j} \left| {\hat{u}_{j} - u\left( {s_{j} ,t} \right)} \right|,$$

where $\hat{u}_{i}$ is an approximate solution of option price *s* and *u*_*i*_ is the analytic solution of option price *s*_*i*_.

$$E_{RMS}^{N} = \sqrt {\frac{1}{N}\mathop \sum \limits_{i = 1}^{N} \left( {\hat{u}_{i} - u_{i} } \right)^{2} } .$$

**Table 1:** Numerical results by difference *N* and $\epsilon$ at *t* = 0 for regular nodal points

*N*	$\epsilon$ = 16		$\epsilon$ = 18		$\epsilon$ = 20	
	$E_{\infty }^{N}$	$E_{RMS}^{N}$	$E_{\infty }^{N}$	$E_{RMS}^{N}$	$E_{\infty }^{N}$	$E_{RMS}^{N}$
8	1.0495E−001	3.3522E−002	1.1481E−001	3.6269E−002	1.2269E−001	3.8477E−002
16	8.7267E−002	2.6660E−002	1.0034E−001	3.0026E−002	1.1138E−001	3.2850E−002
32	3.4618E−002	1.3624E−002	5.3060E−002	1.9251E−002	6.5506E−002	2.2310E−002
64	2.2827E−003	1.7732E−004	2.3082E−003	2.1776E−004	2.7850E−003	2.6605E−004
128	1.9114E−003	4.7497E−005	1.9118E−003	4.9024E−005	1.9124E−003	5.1249E−005

Tables 1 and 2 show convergence trends of the present method, with *N* = *K* where *N* and *K* are the number of points in the spatial and temporal domain. From these tabular results one can observe that Crank–Nicolson’s method converges to the exact solution and the maximum error and root mean square error decreases while increasing the number of nodes for any $\epsilon$.

**Table 2:** Numerical results by difference *N* for σ = 0.4, *r* = 0.08 and $\epsilon$ = 16 at *t* = 0 for irregular nodal points

*N*	∆*t* = 0.1		∆*t* = 0.01	
	$E_{\infty }^{N}$	$E_{RMS}^{N}$	$E_{\infty }^{N}$	$E_{RMS}^{N}$
8	1.9276E−002	9.2465E−003	1.9277E−002	9.2453E−003
16	3.9246E−002	9.2326E−003	3.9245E−002	9.2318E−003
32	2.3700E−002	4.6122E−003	2.3713E−002	4.6136E−003
64	2.3812E−003	1.9354E−004	2.3753E−003	1.9002E−004
128	5.0184E−004	2.0978E−005	4.2664E−004	2.0389E−005

### The European call option for long maturity time

We consider the Black–Scholes equation for the European call option with parameters *r*, *E*, *T* with a wide range of volatilities σ and strike price *E*.

**Fig. 6:**
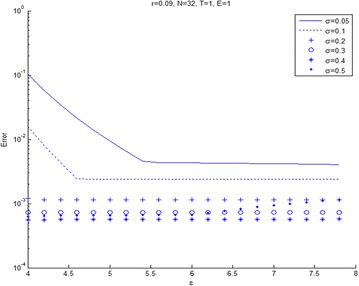
Error plot for European call options with parameters *r* = 0.09, *E* = 1, at maturity time *T* = 1

**Fig. 7:**
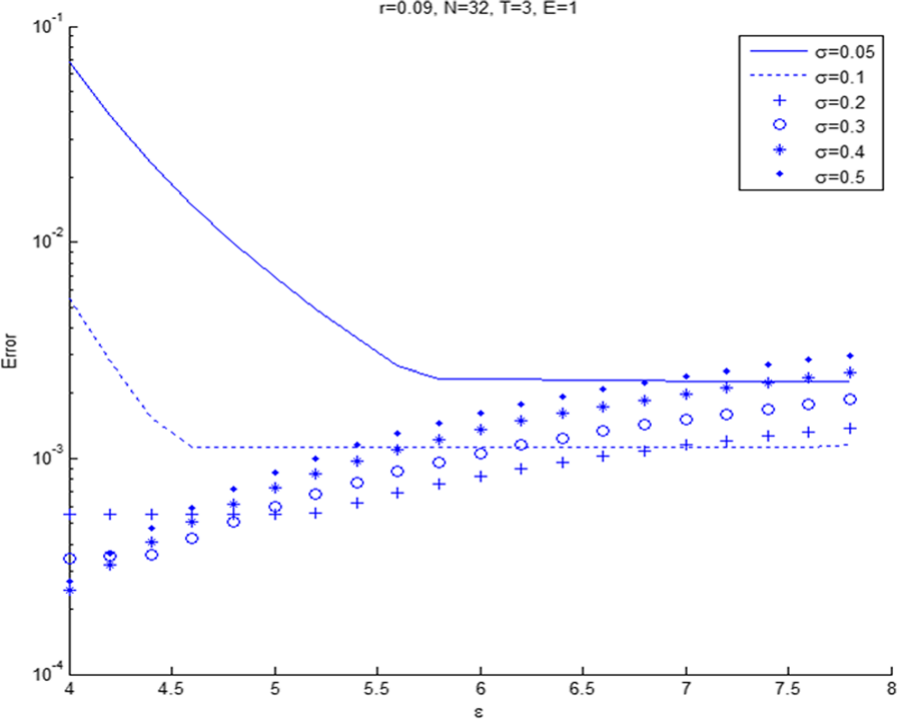
Error plot for European call options with parameters *r* = 0.09, *E* = 1 at maturity time *T* = 3

**Fig. 8:**
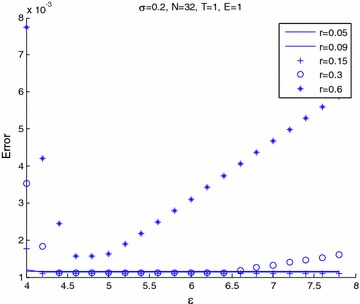
Error plot for European call options with parameters *E* = 1, *T* = 1 for volatility σ = 0.2

**Fig. 9:**
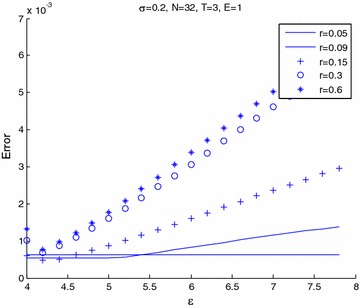
Error plot for European call options with parameters *E* = 1, *T* = 3 for volatility σ = 0.2

Figures 6 and 7 shows the relation between maximum error and $\epsilon$ for varieties of volatility. Figure 6 presents that $\epsilon$ increases and then the error tends to stabilize for any volatility. In Fig. 7, we see that the error will increase when the volatility become small and shape parameter, $\epsilon$ becomes large. The other volatility tends to slowly increase the error. Figures 8 and 9 shows the relation between maximum error and $\epsilon$ for a variety of risk-free interest rates. These figures are observed at large values of risk-free interest rates due to the convection-dominant nature of the problem.

### The European call option with volatility function

We consider the Black–Scholes equation for the European call option with parameters *r* = 0.06, *E* = 1, $T = 1,\sigma (s,\tau ) = 0.15(0.5 + 2\tau )\left( {\frac{{\frac{s}{100} - 1.2)^{2} }}{{\left( {\frac{s}{100}} \right)^{2} + 1.44}}} \right),x_{ \hbox{min} } = - \ln (4E),x_{ \hbox{max} } = \ln (4E).$ The volatility function is the same as the given in Kadalbajoo et al. (2012) and Kumar et al. (2015). In this case, the exact solution is not known. Table 3 shows that the comparison of value options from finite differences, the cubic spline and MLPG method. We found that these results are very similar. Table 4 shows that our value options results differ by only 0.54 % as compared to the finite difference method and 0.36 % as compared to the cubic spline method.

**Table 3:** Approximate solutions of option values by the difference method for *N* = 8, *K* = 8, *t* = 0

Node no.	Finite difference	Cubic spline	MLPG
1	0.0000E+00	0.0000E+00	0.0000E+00
2	3.1998E−04	−1.8384E−04	1.2871E−03
3	4.5990E−03	−2.3873E−03	−7.8427E−04
4	4.7344E−02	4.2946E−02	4.6467E−02
5	2.9821E−01	2.9823E−01	3.0188E−01
6	8.7309E−01	8.6679E−01	8.6843E−01
7	1.7531E+00	1.7490E+00	1.7509E+00
8	3.0582E+00	3.0582E+00	3.0582E+00

**Table 4:** Percentage of difference in value options by the MLPG with other methods for *N* = 8, *K* = 8, *t* = 0

Node no.	Finite difference	Cubic spline
1	0.00	0.00
2	0.10	0.15
3	0.54	0.16
4	0.09	0.35
5	0.37	0.36
6	0.47	0.16
7	0.22	0.19
8	0.00	0.00

## Conclusion

In this paper, the MLPG method was proposed for the Black–Scholes equation, which is transformed for a non-degenerate partial differential equation, and used moving kriging shape functions which have Kronecker delta properties. The temporal discretization was chosen by the Crank–Nicolson method. The eigenvalue (*λ*) of *B* depends on the mesh spacing parameter (*h*) and the shape parameter ($\epsilon$). The relation between *Re*(*λ*)_*max*_ and the volatility (σ) is an increasing function for all shape parameters. The relation between *Re*(*λ*)_*max*_ and risk free interest rates (*r*) tends to decrease functions for all shape parameters. If the mesh length (*h*) increases, then the smallest of shape parameters $\epsilon$ will be decreased. The numerical results have demonstrated the accuracy and efficiency of the present methods. The present method gives the value option in both regular and irregular nodal points. We found that the relation between errors and shape parameters vary by volatility and risk-free interest rates. This method works well for finding the approximate solution of option pricing with a volatility function.

## References

[CR1] Atluri N, Shen S (2002). The meshless local Petrov–Galerkin (MLPG) method: a simple and less-costly alternative to the finite element and boundary element methods. CMES.

[CR2] Bastian-Pinto CL (2015). Modeling generic mean reversion processes with a symmetrical binomial lattice—applications to real options. J Procedia Comput Sci.

[CR3] Bjork T, Hult H (2005). A note on Wick products and the fractional Black–Scholes model. Finance Stoch.

[CR4] Black F, Scholes M (1973). The pricing of options and corporate liabilities. J Polit Econ.

[CR5] Cen Z, Le A (2010). A Robust finite difference scheme for pricing American put options with singularity-separating method. Numer Algorithms.

[CR6] Cen Z, Le A (2011). A robust and accurate finite difference method for a generalized Black–Scholes equation. J Comput Appl Math.

[CR7] Cen Z, Le A, Xu A (2013). Finite difference scheme with a moving mesh for pricing Asian options. J Appl Math Comput.

[CR8] Cox JC, Ross S, Rubinstein M (1979). Option pricing: a simplified approach. J Financ Econ.

[CR9] Glazyrina A, Melnikov A (2016). Bernstein’s inequalities and their extensions for getting the Black–Scholes option pricing formula. J Stat Probab Lett.

[CR10] Huang J, Cen Z (2014). Cubic spline method for a generalized Black–Scholes equation. Math Probl Eng.

[CR11] Kadalbajoo MK, Tripathi LP, Kumar A (2012). A cubic B-spline collocation method for a numerical solution of the generalized Black–Scholes equation. Math Comput Model.

[CR12] Kleinert K, Korbel J (2016). Option pricing beyond Black–Scholes based on double-fractional diffusion. J Phys A.

[CR13] Kumar S, Kumar D, Singh J (2014). Numerical computation of fractional Black–Scholes equation arising in financial market. Egypt J Basic Appl Sci.

[CR14] Kumar A, Tripathi LP, Kadalbajoo MK (2015). A numerical study of Asian option with radial basis functions based finite differences method. Eng Anal Bound Elem.

[CR15] Lesmana DC, Wang S (2013). An upwind finite difference method for a nonlinear Black–Scholes equation governing European option valuation under transaction costs. J Appl Math Comput.

[CR16] Mohammadi R (2015). Quintic B-spline collocation approach for solving generalized Black–Scholes equation governing option pricing. Comput Math Appl.

[CR17] Moon KS, Kim H (2013). An adaptive averaging binomial method for option valuation. J Oper Res Lett.

[CR18] Phaochoo P, Luadsong A, Aschariyaphotha N (2016). The meshless local Petrov–Galerkin based on moving kriging interpolation for solving fractional Black–Scholes model. J King Saud Univ Sci.

[CR19] Schwartz E (1977). The valuation of warrants: implementing a new approach. J Financ Econ.

[CR20] Song L, Wang W (2013). Solution of the fractional Black–Scholes option pricing model by finite difference method. J Abstr Appl Anal.

[CR21] Vazquez C (1998). An upwind numerical approach for an American and European option pricing model. Appl Math Comput.

[CR22] Yimnak K, Luadsong A (2014). A local integral equation formulation based on moving kriging interpolation for solving coupled nonlinear reaction–diffusion equations. Adv Math Phys.

[CR23] Zhang H, Liu F, Turner I, Chen S (2016). The numerical simulation of the tempered fractional Black–Scholes equation for European double barrier options. J Appl Math Model.

